# Device Postannealing with Superficially Ag‐Modified Absorber for High‐Efficiency Cd‐Free Cu_2_ZnSnS_4_ Solar Cells

**DOI:** 10.1002/smsc.202500545

**Published:** 2026-02-21

**Authors:** Xiaojie Yuan, Jialiang Huang, Jianjun Li, Kaiwen Sun, Ao Wang, Xiaojing Hao

**Affiliations:** ^1^ School of Photovoltaic and Renewable Energy Engineering University of New South Wales Sydney Australia; ^2^ Shenyang National Laboratory for Materials Science Institute of Metal Research Chinese Academy of Sciences Shenyang China

**Keywords:** carrier collection, defect engineering, device annealing, grain growth, kesterite solar cells

## Abstract

Cation substitution is a promising research direction for further improving the device performance of Cu_2_ZnSnS_4_ (CZTS) solar cells by mitigating recombination loss at deep‐level defects. Here, a superficial Ag_2_ZnSnS_4_ (AZTS) modification process on presulfurized cosputtered CZTS precursor is employed for fabricating a CZTS absorber film of layer‐spanning grains with reduced interfacial Cu_Zn_ tail states of deeper level and increased minority carrier lifetime. Results show that the open‐circuit voltage and short‐circuit current density of the CZTS device are increased after the modification, though the FF is decreased due to a reduced carrier density of CZTS and a degraded junction electric field. With a postdevice air annealing process, the poor junction quality of the AZTS‐modified CZTS can be recovered without introduction of extra interfacial deep‐level defects. An encouraging efficiency of 10.50% with a remarkably high short‐circuit current density of 22.9 mA cm^−2^ is achieved with antireflecting coating. This study demonstrates a unique method for reducing interfacial and bulk recombination loss while improving the junction quality of CZTS, which can help in designing strategies for improving the *V*_OC_ and FF of CZTS.

## Graphical abstract

A superficial Ag‐modification process using spin‐coated Ag_2_ZnSnS_4_ layer on presulfurized CZTS precursor is proposed to improve the surface defect properties. The absorber fabrication process reduces the interfacial nonradiative recombination loss and suppresses the generation of deep defects during device annealing, enabling 10.53% efficient Cd‐free CZTS solar cells.

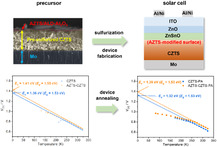

## 1 Introduction

Nontoxic kesterite CZTS has become a potential and attractive alternative to Cu(In, Ga)Se_2_ (CIGS) and CdTe solar cell material due to its abundant elemental source in the earth's crust [1]. Although the certified highest device conversion efficiencies of CZTSSe and CZTS have already been reported at 15.8% and 13.2%, respectively [2], they are still far behind commercialized CIGS solar cells (23.6%) and the Shockley–Queisser efficiency limit of around 32% [3]. The large open circuit voltage (*V*_OC_) deficit is commonly considered to be one of the key factors that limit the efficiency of CZTS [4, 5, 6]. Although the reasons for the large *V*_OC_ deficit are not so clear, it is commonly believed that *V*_OC_ of CZTS device can easily be impeded by the interfacial recombination caused by high density of superficial acceptor defects on CZTS grains [7] or nonoptimal band alignment between CZTS absorber and buffers [8]. One important culprit for the low *V*_OC_ is the randomly distributed Cu–Zn disorder in the whole CZTS absorber [9], which can introduce a high concentration of Cu_Zn_ acceptor defects near the junction interface, pinning the Fermi level near the mid‐gap region of CZTS and deteriorating the quasi‐Fermi level splitting [10, 11]. Therefore, investigating strategies to control and reduce the formation of Cu_Zn_ acceptor defects can play a critical role in breaking the bottleneck of improving *V*_OC_.

The formation of Cu_Zn_ acceptor defects in CZTS mainly originates from the low formation energy of Cu_Zn_ antisite defects since Cu and Zn have similar atomic number and size [12, 13]. One effective strategy to mitigate the Cu_Zn_ acceptor defects in CZTS is to introduce Ag into the M^I^ site of CZTS. This method can help suppress the formation of M^I^/M^II^ antisite defects since Ag_Zn_ antisites have much higher formation energy than Cu_Zn_ [11]. ACZTS and ACZTSSe solar cells with over 11.2% and 13.0%, respectively, have been demonstrated, both demonstrating reduced Urbach energy and Cu_Zn_ antisites, enlarged grain sized and thus lower *V*_OC_ deficit after Ag introduction [14, 15]. Additionally, a high Ag substitution with Ag/(Ag + Cu) > 0.5 can result in the formation of n‐type kesterite (Ag, Cu)_2_ZnSnS_4_ (ACZTS) or (Ag, Cu)ZnSnSe_4_ (ACZTSe) [16, 17], which is beneficial to construct large band bending and type inversion at the interface between CZTSSe and buffer layer. Nevertheless, the commonly employed Ag substitution amount is usually not large enough to achieve type‐inversion at CZTSSe surface [18, 19, 20]. Such slight Ag substitution is also beneficial to device performance, as the bulk ACZTS tends to transform into defective stannite phase and increase interface recombination when Ag/(Ag + Cu) > 0.2 [21]. On the other hand, heavy Ag substitution at the surface of CZTSSe has been proven to effectively eliminate Cu_Zn_ acceptor defects and construct stronger band bending in CZTSSe for further *V*_OC_ improvement [22, 23]. Methods such as postselenization with an ultrathin AgF layer on the surface of CZTSSe film [23], and deposition of an Ag_2_ZnSnS_4_ (AZTS) layer onto Al_2_O_3_‐capped solution‐processed amorphous CZTS precursor film [22], have successfully produced highly Ag‐substituted CZTSSe surface layer with Ag/(Ag + Cu) ratios of around 20% and over 50%, respectively. However, these surface Ag substitution methods did not address the commonly observed carbon‐rich fine‐grain layer, nor did they improve carrier collection efficiency at the back interface. In addition, the role of bottom‐layer crystallinity remains unclear. Moreover, surface Ag substitution has not been applied to pure sulfide CZTS, and suppressing the fast Ag diffusion is challenging for CZTS as the processing temperature is higher than CZTSSe [24]. Hence, a systematic study on surface Ag substitution for layer‐spanning CZTS has great potential to boost the device performance of kesterite CZTS solar cells.

In this work, we successfully obtain a CZTS absorber with both large grains spanning through the entire film thickness and superficial Ag substitution by employing a hybrid cosputtering/spin‐coating strategy for precursor fabrication. Investigations show that a cosputtered precursor can avoid the formation of a carbon‐rich fine‐grain layer in the final film, while a spin‐coated thin AZTS layer on top can achieve superficial Ag substitution and enlargement of grain size. To suppress the fast diffusion of surface Ag into CZTS bulk, the cosputtered film is presulfurized into crystalized CZTS. The effect of this initial crystallinity on the subsequent grain growth process is investigated. Meanwhile, the effect of an ultrathin Al_2_O_3_ barrier layer on the presulfurized CZTS by atomic layer deposition (ALD) is also studied. The devices with the layer‐spanning Ag‐modified CZTS large grains not only achieve high carrier collection efficiency near the back contact but also demonstrate reduced recombination loss via Cu_Zn_ tail states of deeper level at the junction interface as well as in the bulk. Furthermore, the reduced interfacial recombination also helps maintain a high‐quality junction interface and device performance with device air annealing, without generating extra interfacial defects. This has also resolved the problem of degraded interface quality with device air annealing in our previous work [25]. Overall, this study provides a unique strategy to obtain layer‐spanning CZTS large grains with enhanced carrier collection efficiency and reduced superficial tail states via surface Ag substitution.

## 2 Results and Discussions

### 2.1 Construction of AZTS‐Modified CZTS Absorber Layer

**FIGURE 1:**
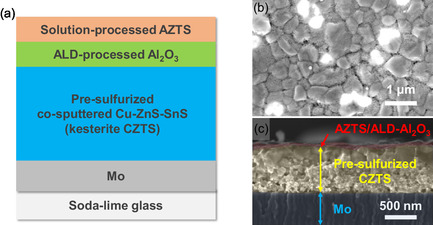
(a) Schematical diagram of the modified CZTS precursor structure, (b) top‐view and (c) cross‐sectional view SEM images of the typical precursor film.

The Ag‐modified CZTS absorber layer is fabricated by sulfurizing a spin‐coated AZTS/ALD‐Al_2_O_3_/presulfurized CZTS/Mo (AZTS‐a‐CZTS) layered precursor. Figure 1a gives the detailed construction process of the precursor film. An optimized presulfurization process at around 420°C for 2 min, where the kesterite CZTS phase starts to form (Figure S1), is employed to transform the cosputtered Cu/ZnS/SnS precursor into mildly crystalized and relatively flat CZTS film (Figure 1b). This flat CZTS film can support the formation of relatively uniform AZTS nanocrystal top layer (~30 nm thick) compared to the film with lower presulfurization temperature where considerable bumps and valleys are formed (Figure S2b). To tentatively suppress the intense interaction between the AZTS top layer and the presulfurized CZTS film, Al_2_O_3_ is deposited on the presulfurized CZTS film using four cycles of ALD before the deposition of AZTS layer. All the precursor films, including presulfurized CZTS film only (CZTS), CZTS film with AZTS (AZTS‐CZTS), and CZTS film with both ALD‐Al_2_O_3_ and AZTS (AZTS‐a‐CZTS), undergo a high‐temperature sulfurization process at 530°C to prepare the absorber. Here, the ramping rate of the sulfurization process is determined by the phase transition properties of the AZTS top layer. A high ramping rate of 1°C/s at 250°C–530°C is used to suppress the formation of detrimental stannite AZTS phases at around 310°C, phase segregation at 430°C–460°C and Sn loss of AZTS at 400°C–430°C (Figure S3 and Table S1), while a slow ramping rate of 0.17°C/s before 250°C is employed to avoid quick loss of *S* and keep a relatively high *S* partial pressure during the ramping process.

**FIGURE 2:**
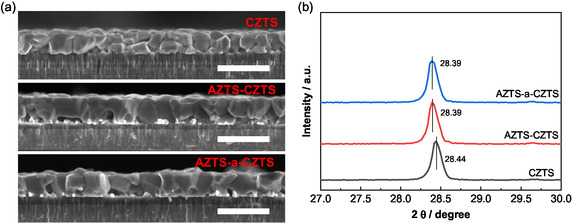
(a) Cross‐sectional view SEM images of sulfurized CZTS, AZTS‐CZTS, and AZTS‐a‐CZTS films. The unit of the scale bar is 1 μm. (b) XRD patterns of the sulfurized CZTS, AZTS‐CZTS, and AZTS‐a‐CZTS films with corresponding (112) diffraction peak positions.

After the sulfurization process, the AZTS‐CZTS and AZTS‐a‐CZTS sample show similar grain sizes (with a final Ag/(Ag + Cu) of around 3%, shown in Table S2), which are significantly larger than the CZTS sample with presulfurization only (Figure 2a). This indicates that the AZTS coating can facilitate the grain growth of the presulfurized CZTS film, and a relatively thin ALD–Al_2_O_3_ layer of four cycles do not hinder the grain growth near the top of the film (eight ALD cycles and over induce small surface grains, Figure S4). The improved grain growth with the AZTS layer can be explained by the introduction of a lower‐melting‐point Ag–S liquid phase (compared with Cu–S), which promotes the grain merging during the high‐temperature stage of sulfurization [18]. Also, the introduced ALD‐Al_2_O_3_ layer can suppress the voids formation by reducing the Sn loss as volatile SnS_*x*_ from the bottom of the absorber (Table S2) [26, 27], which has also been reported in the case of CZTSSe [28]. However, the AZTS‐CZTS and AZTS‐a‐CZTS samples show the same (112) diffraction peak shift toward lower angle compared to reference CZTS in the X‐ray diffraction (XRD) patterns (Figure 2b), indicating the ALD‐Al_2_O_3_ layer does not change the percentage of Ag‐substituted Cu in the CZTS absorber. While we can confirm that the ALD‐Al_2_O_3_ layer stays at the surface of CZTS absorber and the Ag distribution is vertically uniform after the sulfurization (Figure S5), we believe that the Al_2_O_3_ layer could not effectively hinder the Ag diffusion from the AZTS top layer into the presulfurized CZTS. The reason for the inability of Ag blocking effect is probably the ultrathin (several angstroms) and loose nature of the amorphous ALD‐Al_2_O_3_ film, since even a compact TiN barrier layer has been reported to require a thickness of a least 5 nm to block Ag diffusion at 400°C [29]. This means the ultrathin ALD‐Al_2_O_3_ used during sulfurization could not provide an Ag blocking effect as effective as that reported for low‐temperature selenization with ALD‐Al_2_O_3_.

**FIGURE 3:**
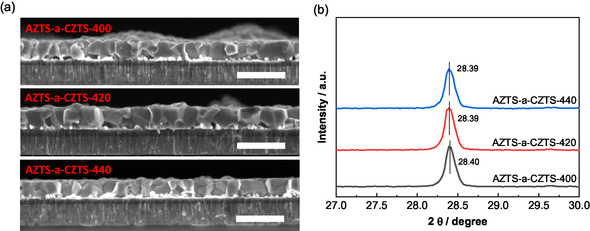
(a) Cross‐sectional view SEM images of the sulfurized AZTS‐a‐CZTS films with different presulfurization temperature for the cosputtered Cu–ZnS–SnS precursor: 400°C, 420°C, and 440°C. The unit of the scale bar is 1 μm. (b) XRD patterns of the three AZTS‐a‐CZTS films with corresponding (112) diffraction peak positions.

The effect of presulfurization temperature of the bottom CZTS layer on the grain growth with AZTS layer is also investigated by employing temperatures at 400°C, 420°C, and 440°C. As shown in Figure 3a, a lower presulfurization temperature at 400°C increases the Sn loss (Table S3) and bottom voids of the final film, as the SnS is not completely immobilized and transformed into crystallographic kesterite CZTS phases (Figure S1). It is worth noting that the increased crystallinity of the presulfurized CZTS at 440°C (Figure S1d) could not suppress the fast diffusion of Ag, as revealed by the same (112) XRD peak shift with varied presulfurization temperatures (Figure 3b). Although the increased crystallinity of the presulfurized CZTS at 440°C can reduce the Sn loss (Table S3) and formation of bottom voids during the final sulfurization, the absorber fails to grow into layer‐spanning films, and small grains and horizontal grain boundaries appear. This means the mass transport between the as‐grown large grains is suppressed when the crystallinity relatively high at 440°C presulfurization process. As a result, a higher presulfurization temperature reduces the carrier collection efficiency and thus lowering device performance (Figure S6 and Table S4). Besides, samples with presulfurization at 400°C and 440°C show lower photoluminescence (PL, 525 nm excitation) energy (Figure S7), more recombination loss at p‐n junction (higher *J*_02_ in Table S4) and higher *V*_OC_ deficits than the 420°C presulfurized sample. While this PL signal mainly reflect on the recombination properties near the surface, we can believe that a lower deep‐level defect density near the surface can be obtained with a medium presulfurization temperature. In this sense, a medium presulfurization temperature at 420°C for fabricating mildly crystallized CZTS is the key to achieve both growth of large grains and reduction of superficial deep‐level defects. Hence, samples with 420°C presulfurization are elected for further investigation.

### 2.2 Recombination and Electrical Properties

**FIGURE 4:**
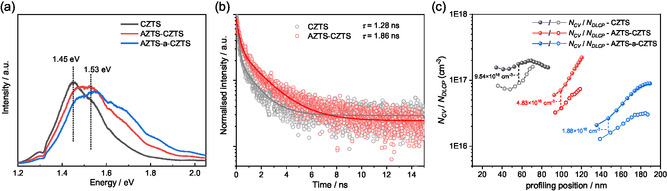
(a) PL spectra (*λ*_ex_ = 525 nm) of the CZTS, AZTS‐CZTS, and AZTS‐a‐CZTS devices, (b) TRPL (*λ*_ex_ = 640 nm) decay of the CZTS and AZTS‐CZTS devices with calculated second‐order lifetime (*τ*) from double exponential function, and (c) carrier densities obtained from the capacitance–voltage profiles (*N*_CV_) and driven level capacity profiles (*N*_DLCP_) of the CZTS, AZTS‐CZTS, and AZTS‐a‐CZTS devices under 10 kHz. The values of acceptor defect density in depletion region are calculated by *N*_CV_‐*N*_DLCP_ at bias voltage of 0 V. Profiling position is the depletion width.

With the AZTS‐modification near the surface of presulfurized precursor, the recombination loss via the deep level defects and tail states near the surface of CZTS absorber are expected to be mitigated. PL spectra with excitation wavelength of *λ*_ex_ = 525 nm, with a penetration depth near the surface of absorber, show a slight blueshift for the AZTS‐CZTS and AZTS‐a‐CZTS samples compared to the CZTS sample, with significant reduced intensity at ~1.45 eV and increased intensity at ~ 1.53 eV (Figure 4a). This change indicates that the proportion of free‐to‐bound radiative recombination via band‐tailed states is reduced near the absorber surface. The nonradiative recombination is qualitatively compared using time‐resolved PL (TRPL). As shown in Figure 4b, the normalized TRPL decay curves can be fitted with a double exponential function with second‐order decay times (*τ*) of 1.28 and 1.86 ns for the CZTS and AZTS‐CZTS samples, respectively. This result suggests that the AZTS‐modification can suppress the nonradiative recombination probably via the improved crystalline quality and the reduction of band‐tailed states. The carrier concentrations at device junction interfaces and near the bulk can be determined by subtracting capacitance–voltage (*C*–*V*) measurement and drive‐level capacitance profile (DLCP, 10 kHz), respectively, with completed devices, as shown in Figure 4c. The AZTS modification reduce the bulk carrier density (*N*_DLCP_) from 8.06 × 10^16^ to 3.76 × 10^16^ cm^−3^, and ALD‐Al_2_O_3_ layer can further reduces the value to 1.60 × 10^16^ cm^−3^ by suppressing the Sn loss as mentioned above [28, 30]. Also, the defect density (*N*_CV_‐*N*_DLCP_ at 0 V) is significantly reduced by half an order from 9.54 × 10^16^ to 4.83 × 10^16^ cm^−3^ and 1.88 × 10^16^ cm^−3^ after the AZTS‐modification without and with ALD‐Al_2_O_3_ layer, respectively, possibly resulting in improved *V*_OC_ and PCE.

### 2.3 Device Performance Analysis

**FIGURE 5:**
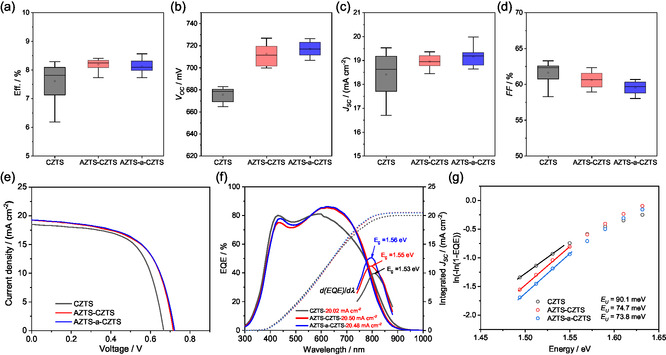
Comparison of the statistical distribution of the device performances of CZTS, AZTS‐CZTS, and AZTS‐a‐CZTS samples: (a) efficiency, (b) *V*_OC_, (c) *J*_SC_, and (d) *FF*. 12 cells from the three samples are measured using cell's total area. (e) Representative *J*–*V* curves and (f) EQE response of the three samples. The corresponding bandgaps are derived from d(EQE)/d ***λ*** with integral *J*_SC_ in (f). (g) Urbach energy (*E*_U_) of CZTSSe solar cells calculated from the EQE spectra in (f).

The statistical device performance and current density–voltage (*J*–*V*) curves of the representative reference CZTS (with presulfurization), AZTS‐CZTS and AZTS‐a‐CZTS devices are shown in Figure 5a–d and Figure 5e, and the corresponding calculated electronic parameters of the devices are also provided in Table 1. Noticeably, the samples with solely AZTS top layer shows an increased power conversion efficiency (PCE) from 7.8% to around 8.3% with narrow distribution compared to the CZTS sample. The improvement is mainly contributed by the increased *V*_OC_ of around 30–40 mV to 711 mV and *J*_SC_ of almost 0.5 mA cm^−2^ though compensated by the slightly decreased *FF* from 63% to around 61% with slightly reduced hole density (Figure 4c). It is also worth mentioning that a thicker AZTS layer (via two coating cycles) can further decrease the FF and the *V*_OC_ (Table S5). The *J*_SC_ increase is attributed to the enhanced external quantum efficiency (EQE) response in the long wavelength region of 600–800 nm (Figure 5f), corresponding to the improved carrier collection efficiency in the bulk. This improvement is associated with the enlarged depletion width (*W*_d_) and improved minority carrier lifetime with Ag‐alloying (Figure 4b). The significant increase of *V*_OC_ can be attributed to the reduced reverse saturation current (*J*_0_) due to the supressed current leakage. This suppression can be explained by the reduced interfacial recombination loss via less interfacial acceptor defects [31] and enhanced effective minority carrier lifetime in the absorber (Figure 4). The reduced interface recombination can be attributed to the suppression of deep‐level defects after the Ag‐alloying, as revealed by the PL spectra (Figure 4a). The decrease in band tailing is also supported by the slightly reduced Urbach energy values (*E*_U_), estimated from the EQE spectra, with *E*_U_ decreasing from 90.1 meV for CZTS to 74.7 meV for AZTS‐CZTS (Figure 5g). Here, the *E*_U_ is calculated from a plot of ln[–ln[1‐EQE]] versus *hν*, where the slopes 1/*E*_U_ are linearly fitted within the photon energy slightly below the bandgap [32] according to the Equation in Note S1. A lower *E*_U_ indicates fewer sub‐bandgap states near the junction interface, which is beneficial for reducing recombination loss and improving *V*_OC_. The slightly reduced *FF* with AZTS modification is attributed to the deteriorated diode‐rectifying characteristics, as reflected by an increased diode ideal factor (*A*), even though the series resistance (*R*_s_) is reduced due to better carrier transport with large grains. This higher *A* value is possibly due to the reduced carrier concentration near the junction induced by Ag‐alloying [33], which weakens the junction electric field and thus leads to lower carrier collection efficiency near the junction, as confirmed by the reduced EQE response in the short wavelength region (Figure 5f).

**TABLE 1:** Photovoltaic parameters of the representative CZTS, AZTS‐CZTS, and AZTS‐a‐CZTS samples.

	*J*_SC_, mA cm^−2^	*V*_OC_, mV	*FF*, %	Eff., %	*R*_sh_, Ω cm^2^	*R*_s_, Ω cm^2^	*A*	*J*_0_, A cm^−2^	*E*_*g*_, eV	*E*_*g*_ */q‐V*_OC_, V
CZTS	18.46	666	63.28	7.78	695	1.27	2.04	9.70 × 10^−6^	1.53	0.863
AZTS–CZTS	18.95	711	61.15	8.24	694	0.57	2.58	5.97 × 10^−6^	1.55	0.839
AZTS‐a‐CZTS	19.25	722	60.04	8.35	554	0.80	2.80	6.23 × 10^−6^	1.56	0.838

In contrast, although the AZTS‐a‐CZTS sample shows a comparable device performance to the AZTS–CZTS sample with slightly lower *E*_U_ (73.8 meV), higher *V*_OC_ (722 mV) and *J*_SC_, the PCE is limited by the further lower *FF* (60%). This lower *FF* is caused by the poorer junction quality (*A* value of ~2.80), which can be explained by the even lowered carrier concentration of CZTS (Figure 4c) due to the suppressed Sn loss (Table S2) and decreased (Ag + Cu)/(Zn + Sn) molar ratio [30]. Besides, the increase of Sn content can also account for the slightly increased *E*_g_ to 1.57 eV [34] and slightly shift the main PL peak to higher energy (Figure 4a). In this sense, the functions of the introduced ALD‐Al_2_O_3_ layer between AZTS and prsulfurized CZTS are suppressing the Sn loss and reducing the carrier concentration of CZTS rather than limiting the Ag migration from AZTS top layer to presulfurized CZTS films.

### 2.4 Defect Property Analysis

**FIGURE 6:**
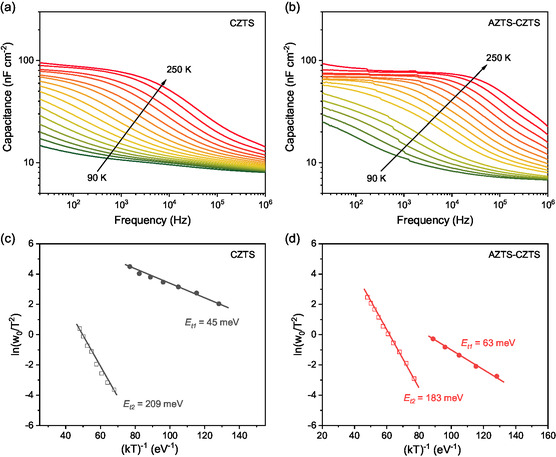
Temperature‐dependent *C*–*f* spectra obtained from admittance spectroscopy for the (a) CZTS device and (b) AZTS‐CZTS device. Arrhenius plots obtained from corresponding admittance spectra: (c) CZTS device and (d) AZTS‐CZTS devices. The *E*_*t*_ is derived from the slop of the linear fitting.

The change of defect property with AZTS‐modification was further investigated by measuring the temperature‐dependent admittance spectroscopy (AS) from 90–250 K. Figure 6a,b shows the AS results of CZTS and AZTS‐CZTS, respectively, and Figure 6c,d display the Arrhenius plots from which the defect activation energy (*E*_t_) can be extracted according to the following equation [35]

$$\omega_{0}=2\nu_{0}\exp\left(-\frac{E_{t}}{kT}\right)$$

where *k* is the Boltzmann constant, and *ω*_0_ is the inflection angular frequency of the electronic transition, determined by the maxima of *fdC*/*df*, and *ν*_0_ the attempt‐to‐escape frequency of the defects. Two defect levels can be identified from the temperature‐dependent *C*–*f* spectra. A pronounced capacitance step at high temperature indicates the presence of a dominant deep‐level defect, while a smaller step appearing at low temperature is attributed to a shallow defect state. The activation energy (*E*_t_) extracted from the Arrhenius analysis of the deep‐level defect decreased from 209 to 183 meV after the AZTS modification, though the *E*_t_ of shallow defect slightly increased from 45 to 63 meV. According to the literature, the deep‐level defect in the range of 0.12–0.25 eV can be notified as a Cu_Zn_ tail states [13]. In this sense, the decreased *E*_t_ indicates that the AZTS modification can reduce the Cu_Zn_ tail states of deeper level, which has also been reported in other reported Ag‐modified kesterite [14, 22, 23]. The reduction of deep‐level tail states is also supported by the smaller *E*_U_ of AZTS–CZTS (74.7 meV) than CZTS (90.1 meV) as shown in Figure 5g. The reduced *E*_t_ can echo the reduced nonradiative recombination loss at junction interface via deep‐level defects, contributing to the *V*_OC_ increase.

### 2.5 Device Performance Analysis with Device Annealing

Further enhancing the performance of AZTS‐modified CZTS can be achieved by improving the junction quality and carrier collection efficiency near the junction interface. It is widely reported that the postair annealing processes using CdS‐buffered CZTS absorber or device at around 300°C can improve the junction quality and reduce nonradiative recombination in the junction interface and bulk absorber via Cd diffusion from the CdS layer to bulk absorber [36, 37, 38]. However, for ZnSnO‐buffered CZTS device, the optimal device annealing temperature is much lower at around 210°C–225°C according to our published results, as a higher annealing temperature can cause severe shunting problems [25, 39]. The function of our device air annealing process is enhancing the carrier concentration of the absorber and the junction quality by removing the charge‐compensating Na_i_^+^ shallow donor defects within the absorber layer [25]. This moderate device air annealing temperature can also avoid the penetration of Na from CZTS to ZnSnO buffer layer (~10 nm) that could create shunting paths and damage the p–n junction.

**FIGURE 7:**
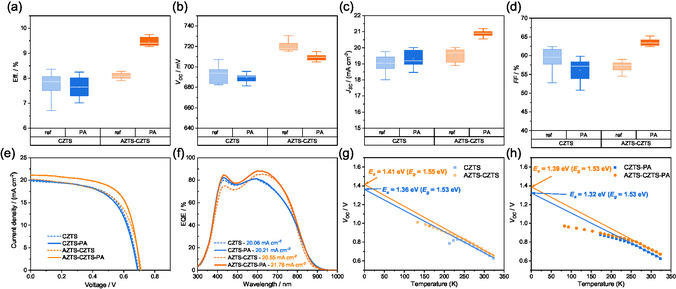
Comparison of the statistical distribution of the device performances of CZTS and AZTS–CZTS samples with and without PA process: (a) efficiency, (b) *V*_OC_, (c) *J*_SC_, and (d) *FF*. 12 cells from the three samples are measured using cell's total area. (e) Representative *J*–*V* curves and (f) EQE response of the CZTS and AZTS–CZTS samples with and without PA process. The corresponding bandgaps are derived from d(EQE)/d ***λ*** with integral *J*_SC_ in (f). Plot of *V*_OC_‐*T* and the linear fit (263–323 K) of the recombination activation energy *E*_a_ for CZTS and AZTS–CZTS devices (g) without and (h) with PA process.

To further improve the junction quality of the AZTS‐CZTS and AZTS‐a‐CZTS device, we adopted the device air annealing process at 220°C for 3 min as reported in our previous work [25]. This process is expected to improve the carrier collection efficiency near the junction area and thus increasing the *J*_SC_ and *FF*. The statistical device performance and *J*–*V* curves of the representative reference CZTS (with presulfurization), AZTS‐CZTS devices before and after device postannealing (PA) process are shown in Figure 7a–d and Figure 7e, and the corresponding calculated electronic parameters of the devices are also provided in Table 2. Noticeably, the AZTS–CZTS samples show significant PCE improvement from 8.3% to around 9.5% after the PA process, with remarkable average increases of *J*_SC_ from ~19.6 to ~21.0 mA cm^−2^, and *FF* from 58% to 63%. The *J*_SC_ increase is contributed by the significant improved EQE response in the short wavelength region of 400–600 nm (Figure 7f). The *FF* enhancement is from the improved diode‐rectifying characteristics and charge transport corresponding to the markedly reduced *A* factor (from 2.72 to 2.26) and increased shunt resistance (*R*_sh_). Although the *V*_OC_ is slightly reduced due to the reduction of *E*_g_, the *J*_0_ and *E*_g_ */q‐V*_OC_ values slightly decrease, indicating the interface recombination loss and leakage current are also suppressed after the PA process. The AZTS‐a‐CZTS sample also shows a similar increase of *J*_SC_ and *FF* with PA, but the final average performance is lower than the AZTS–CZTS sample with PA (Figure S8). This lower performance is related to the poorer p–n junction quality (*A* = 2.88) and lower *FF* (Table S6) possibly induced by too low initial carrier concentration, with which limited increase of carrier concentration can be obtained after PA process. By contrast, the PCE of the CZTS sample drops by 0.4% in average after the PA process with the slightly reduction of *V*_OC_ and *FF*. The degraded device performance is attributed to the increased leakage current near the junction interface corresponding to the tripled *J*_0_ and reduced *R*_sh_. This result can align with our previous finding that sample with relatively high Cu/Sn ratio in precursor (~1.85) fails to gain universal improvement on *V*_OC_ and *FF* via the PA process [25], which can be explained by reduced Na extraction from glass substrate (at high Cu content) for passivation of interfacial deep‐level acceptor defects. Interestingly, the AZTS‐CZTS sample still shows relatively high *V*_OC_ and enhanced *J*_SC_ and *FF* with a relatively high (Ag + Cu)/Sn ratio of around 1.85 in precursor. This indicates that the AZTS modification can change the interfacial defect evolution during the PA process and improve the defect properties near the junction interface even at a high (Ag + Cu)/Sn ratio.

**TABLE 2:** Photovoltaic parameters of the representative CZTS and AZTS–CZTS samples with and without postdevice annealing (PA).

	*J*_SC_, mA cm^−2^	*V*_OC_, mV	*FF*, %	Eff., %	*R*_sh_, Ω cm^2^	*R*_s_, Ω cm^2^	*A*	*J*_0_, A cm^−2^	*E*_g_, eV	*E*_g_ */q‐V*_OC_, V
CZTS	19.76	695	61.99	8.51	680	1.25	2.43	7.80 × 10^−6^	1.53	0.835
CZTS‐PA	20.01	688	59.87	8.25	566	1.09	2.68	2.27 × 10^−5^	1.53	0.842
AZTS–CZTS	20.27	713	59.26	8.56	379	0.45	2.72	3.20 × 10^−6^	1.55	0.837
AZTS–CZTS‐PA	21.19	705	65.25	9.75	759	0.44	2.26	3.14 × 10^−6^	1.53	0.825

The function of AZTS‐modification on improving the interface quality with PA process is further confirmed by the analysis of activation energy (*E*_a_) derived from temperature‐dependent *V*_OC_. As shown in Figure 7g, the AZTS–CZTS sample shows a much higher *E*_a_ of 1.41 eV compared to 1.36 eV for the CZTS sample before the PA process, along with a smaller activation energy deficit (*E*_g_‐*E*_a_). This suggests reduced recombination losses via interfacial deep‐level defects, consistent with the earlier discussion. After the PA process, the *E*_a_ of the AZTS–CZTS sample slightly drops to 1.39 eV and the activation energy deficit is not changed, while the *E*_a_ for CZTS sample drops sharply to 1.32 eV with larger activation energy deficit (Figure 7h). This indicates that the PA process with AZTS‐modification does not introduce extra recombination centers at junction interface, which is different from the typical PA process with pure CZTS. Here, the positive effects by In diffusion from the ITO layer into the junction interface area can be excluded according to our previous results, where no detectable In increase could be found in the buffer layer and the absorber layer at this low annealing temperature [25]. In a typical PA process, the carrier concentration of the CZTS and junction quality can be improved by removing the acceptor‐compensated Na_i_^+^ donor defects in the bulk and junction interface [25]. Nevertheless, the PA process also degrades the interface quality as the residual Na at the junction interface is not enough for passivating the Cu_Zn_ tail states. In this sense, the initial halved interfacial defect concentration with the AZTS‐modification (4.83 × 10^16^ cm^−3^) compared to that of the reference CZTS samples (9.54 × 10^16^ cm^−3^) is believed to contribute to the formation of less interfacial defects during the PA process. In addition, the AZTS‐modification has also decreased the activation energy *E*_t_ of Cu_Zn_ tail states by 26 meV at the junction interface (Figure 6). This can effectively reduce the contribution of tail states of deeper level at the junction interface and thus enhancing the tolerance for the removal of interface‐passivating Na during the PA process. In other words, the superficial modification with AZTS layer on CZTS precursor can suppress the formation of Cu_Zn_ tail states of deeper level and thus suppressing the interface quality degradation during the PA process.

**FIGURE 8:**
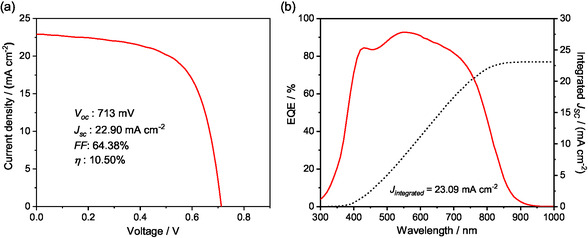
(a) Illuminated *J*–*V* curves (AM 1.5) and (b) EQE curves of the best AZTS‐CZTS‐PA device with ARC. The integrated *J*_SC_ was calculated from EQE and AM 1.5 spectra.

After depositing an ARC on the best AZTS‐CZTS device with PA process, we further achieved a relatively high efficiency at 10.50% for Cd‐free CZTS device (total area). As shown in Figure 8a, the AZTS‐CZTS device with PA process enabled a significantly high *J*_SC_ of 22.90 mA cm^−2^, with *V*_OC_ of 713 mV and *FF* of 64.38%. Figure 8b presents the EQE spectra of the best performing device, with the integrated current density measured notably high at 23.09 mA cm^−2^ under 1 sun illumination at 1000 W m^−2^. This value is slightly higher than the *J*_SC_ obtained from the total‐area *J*–*V* measurement, because the EQE measurement has avoided the optical loss from the metal grid shading on devices. Although the highest EQE has reached 95% at wavelength of 550 nm, the values between 600–750 nm are still lower than 90%. This indicates that the carrier collection efficiency in bulk CZTS and near the rear interface are still low, which is also demonstrated by the short effective minority carrier lifetime of 0.35 ns in the bulk (Figure S9). Further optimization will focus on reducing local detrimental defect density by adjusting the local element composition uniformity during grain growth process (especially within the presulfurized sputtered layer), which is crucial for boosting the bulk carrier lifetime and thus achieving *V*_OC_ and efficiency breakthroughs of CZTS solar cells.

## 3 Conclusion

In summary, this work demonstrates that AZTS surface layer, even without the application of ALD‐Al_2_O_3_ capping layer, places an important role in reducing the deep‐level acceptor defect concentration of CZTS absorber surface and bulk and improving the minority carrier lifetime of the absorber. Mild crystalized CZTS precursor with presulfurization is the key to suppressing the formation of superficial Cu_Zn_ tail states of deeper level while assisting the layer‐spanning grain growth with the AZTS top layer. The strategy of AZTS‐modification on presulfurized CZTS precursor enables the *V*_OC_ boost of 30–40 mV and better carrier collection in the bulk absorber, with slightly reduced *FF* due to the decreased carrier density and junction quality. The decreased *FF* and junction quality can be recovered by applying device PA process, where the charge‐compensational Na_i_^+^ near the junction interface can be removed, without introducing extra interfacial defects. This binary strategy effectively reduces recombination loss at both the junction interface and bulk, achieving a remarkable PCE of 10.5% with a *J*_SC_ of 22.90 mA cm^−2^. These findings confirm the necessity of low concentration of interfacial defects and shallower tail states in CZTS when applying device annealing and provide direction for obtaining high‐performance CZTS solar cells.

## 4 Experimental Section

### 4.1 CZTS Absorber Fabrication

The fabrication procedure of the CZTS absorber layer was modified based on our baseline process using an AZTS/ALD‐Al_2_O_3_/presulfurized CZTS/Mo precursor structure. Typically, cosputtered precursor Cu/ZnS/SnS films with Cu/Sn ratio at 1.65–1.70 and Zn/Sn at 1.10–1.15 on Mo‐coated soda lime glass (SLG) were presulfurized in an rapid thermal processor (AS One 100) at 400°C–440°C for 2 min under sulfur atmosphere (100 mg S) with a ramping rate of 10°C min^−1^ and cooled down to room temperature naturally. The element ratios were measured with inductively coupled plasma mass spectrometry (ICP‐OES, Perkin Elmer, Nexion 5000). Then, an ultrathin Al_2_O_3_ layer was directly deposited on the presulfurized CZTS absorber by ALD method using trimethylaluminum (TMA) and deionized water as precursors, with argon (Ar) as the carrier gas. The ALD process was conducted in the Fiji G2 ALD system (CambridgeNanotech) at a substrate temperature of 150°C, while keeping water and the TMA precursor at room temperature. During the ALD process, Al:Ar:H_2_O:Ar cycle with pulse lengths of 0.06:10:0.06:10 s was repeated for four times to obtain the desired high coverage of Al_2_O_3_ layer on the samples. For comparison, samples without and with 8 and 12 ALD cycles were also prepared.

AZTS top layer was prepared by spin‐coating AZTS precursor solution on the as‐prepared samples followed by heat treatment. The precursor solution was prepared by dissolving Ag(CH_3_COO), Zn(CH_3_COO)_2_, SnCl_4_ and thiourea in *N*, *N*‐Dimethylformamide (DMF) in an N_2_‐filled glovebox with O_2_ and H_2_O below 5 ppm. The corresponding molar ratio was 1.8:1.1:1:7.8 and the [Sn] concentration was 0.12 mol/L. The solution was spin‐coated onto the presulfurized CZTS absorber at a spinning rate of 2000 rpm followed by heat treatment on a hot plate at 275°C for 2 min. Only one cycle of coating‐baking was applied in this process. Finally, the film was sulfurized in a quartz box in a rapid thermal processor (AS One 100) using combined sulfur & SnS atmosphere at 530°C for 8 min at a certain initial pressure (~700 mbar) of nitrogen, with a slow (0.7°C s^−1^) and then fast (1°C s^−1^) ramping rate and was cooled down to room temperature naturally. The amount of S and SnS were 100 and 15 mg, respectively. The details of the sulfurization profile are discussed in the main text.

### 4.2 Device Fabrication

The CZTS devices were fabricated with an architecture of Mo/CZTS/ZTO/i‐ZnO/ITO/Ni/Al/Ni with or without MgF_2_ antireflection coating (ARC), as described in our previous publication [25]. To achieve better band alignment between buffer and absorber layer, a (Zn, Sn)O (ZTO) buffer layer with a thickness of 10 nm was deposited by the ALD method using a Fiji G2 ALD system (Cambridge Nanotech) at a substrate temperature of 150°C. After that, an i‐ZnO layer of 50 nm and an ITO layer of ~210 nm (sheet resistance lower than 30–40 Ω sq^−1^) were deposited using the AJA RF sputtering. Evaporated Ni/Al/Ni patterning with a height of ~ 3 μm was employed as top contact and the final cell area is 0.22–0.23 cm^2^ defined by mechanical scribing. E‐beam (Lesker PVD75) evaporated Ni–Al–Ni patterning fingers with a height of ~ 2 μm were employed as top contact and the MgF2 layer of 91–100 nm (optimized with target wavelength centers of 550 nm) was deposited using thermal evaporation.

### 4.3 Device Postannealing (PA)

The PA processes of the device were conducted in air by placing the finished device (after the deposition of the top contact) on a preheated hotplate at 220°C for 3 min, followed by fast cooling on a room‐temperature ceramic plate in 20 s. All the PA processes were developed immediately after the *J*–*V* measurement.

### 4.4 Characterizations

The microstructure upon the CZTS was measured using a NanoSEM 230 field‐emission scanning electron microscope (FESEM). The *J*–*V* curves were measured using a solar simulator (ABET IV Tester) with AM1.5 G illumination (100 mW cm^−2^) calibrated with a standard Si reference and a Keithley 2400 sourcemeter. The temperature‐dependent measurement (JV‐T) was conducted in a liquid nitrogen cryostat on a stainless‐steel stage. The measured temperature is between 93–313 K with 10–20 K intervals. EQE data were collected using a QEX10 spectral response system (PV measurements, Inc.) calibrated by the National Institute of Standards and Technology (NIST) certified reference Si and Ge photodiodes. The *C*–*V* measurement was carried out using an impedance analyzer at a frequency of 10 kHz with a DC bias voltage sweeping from −1.6 to 0.6 V. Drive‐level capacitance profiling (DLCP) measurement was performed using a Keithley 2400 sourcemeter, with the AC amplitude varied from 10 to 110 mV and the DC bias using an impedance analyzer at a frequency of 10 kHz with a DC bias voltage sweeping from −1.6 to 0.6 V. AS measurements were performed with a Keithley 2400 sourcemeter at frequency of 10^2^ ~ 10^6^ Hz and temperature of 90–250 K. PL spectra were measured using a 1/4 m monochromator (Cornerstone 260) equipped with a silicon charge‐coupled device (CCD) camera. The continuous wave laser (525 nm, 50 mW) was used as the excitation source and the luminescence was detected by the CCD. TRPL measurements were performed on the devices using time‐correlated single‐photon counting (TCSPC) (Microtime200, Picoquant), with an excitation wavelength of 640 nm. The Suns‐*V*_*OC*_ measurement is performed using a decaying light pulse with a full‐width half maximum (FWHM) of ~ 2 ms from a xenon flash lamp, which enables the quasisteady state photovoltage V_OC_ to be measured across the p–n junction in the open‐circuit condition. The light intensity is monitored by a single‐crystal silicon reference cell plotted against *V*_OC_. Dynamic SIMS depth profiles were performed on Ag‐modified CZTS devices without PA. A Cameca IMS 5fE7 instrument was employed in this analysis, and a primary Cs^+^ beam was used for a 180 × 180 μm^2^ region ofthe surface with an analysis area of 33 μm in diameter.

## Supporting Information

Additional supporting information can be found online in the Supporting Information section. **Supporting** **Fig.** **S1:** (a) Raman spectra of presulfurized CZTS films at different temperature for 2 min. The film forms wurtzite CZTS phase at 410°C (326 cm^−1^) and transforms into kesterite phase at over 420°C (335–337 cm^−1^), cross‐sectional view SEM images of presulfurized cosputtered Cu‐ZnS‐SnS precursor at different presulfurization temperatures: (b) 400°C, (c) 420°C and (d) 440°C. The unit of the scale bar is 1 μm. **Supporting** **Fig.** **S2:** top‐view SEM images of AZTS layer coated on (a) Mo and (b) pre‐sulfurized CZTS film at 400°C. **Supporting** **Fig.** **S3:** (a) Raman spectra and (b) XRD pattern of AZTS films on Mo after annealing in N_2_ atmosphere at different temperature. Here we use three layers of AZTS spin‐coated films instead of the single layer employed on pre‐sulfurized CZTS film to increase the signal collection from Raman and XRD measurement. Raman spectra show the peaks of stannite AZTS phases (marked in dashed lines) start to appear at around 310°C during the annealing, while the XRD pattern shows the spin‐coated AZTS suffers from measurable phase segregation (AZTS, ZnS and SnS) at between 430°C and 460°C. (c) typical annealing profile for the sulfurization of CZTS precursor films. **Supporting** **Fig.** **S4:** Cross‐sectional SEM images of sulfurized AZTS‐a‐CZTS films with different cycles of ALD‐Al_2_O_3_: (a) 0, (b) 4, (c) 8, and (d) 12. The unit of the scale bar is 1 μm. The ALD‐Al_2_O_3_ starts to limit the grain growth with 8 cycles by introducing more horizontal grain boundaries near the surface. **Supporting** **Fig.** **S5:** SIMS depth profile of the typical AZTS‐a‐CZTS device with CdS buffer layer. **Supporting** **Fig.** **S6:** Comparison of the statistical distribution of the device performances of AZTS‐a‐CZTS samples with different pre‐sulfurization temperatures for the cosputtered Cu‐ZnS‐SnS precursor at 400°C (400‐AgAl), 420°C (420‐AgAl) and 440°C (440‐AgAl): (a) efficiency, (b) V_OC_, (c) J_SC_, (d) FF. The sample size is 12 cells for both samples which are measured based on total area device. (e) representative J‐V curves and (f) EQE response of the AZTS‐a‐CZTS samples with different pre‐sulfurization temperatures. The corresponding band gaps derived from d(EQE)/dλ and integral J_SC_ are given in Figure S6f. **Supporting** **Fig.** **S7:** PL spectra (*λ*_ex_ = 525 nm) of the reference CZTS and AZTS‐a‐CZTS devices with different pre‐sulfurization temperatures at 400°C (400‐AgAl), 420°C (420‐AgAl) and 440°C (440‐AgAl). **Supporting** **Fig.** **S8:** Comparison of the statistical distribution of the device performances of AZTS‐CZTS and AZTS‐a‐CZTS samples with and without PA: (a) efficiency, (b) V_OC_, (c) J_SC_, (d) FF. The sample size is 12 cells for both samples which are measured based on total area device. **Supporting** **Fig.** **S9:** TRPL (*λ*_ex_ = 640 nm) decay of the AZTS‐CZTS devices after PA process with calculated effective lifetime (*τ*_eff_) from double exponential function. **Supporting** **Table** **S1:** Compared elemental ratios of AZTS films annealed at different temperatures in N_2_ atmosphere. The data is measured by SEM‐EDS. The comparable Ag/Sn ratios at 370°C and 400°C and increased Ag/Sn ratio from 2.045 at 400°C to 2.161 at 430°C indicates that the Sn loss (in form of SnS as depicted in Figure S3b) and decomposition of the AZTS film start at between 400°C and 430°C. **Supporting** **Table** **S2:** Compared elemental ratios of reference and AZTS‐modified CZTS films after sulfurization. The data is measured by ICP‐OES. The lower (Ag + Cu)/Sn ratio of AZTS‐a‐CZTS absorber at 1.896 than that of AZTS‐CZTS absorber at 1.918 indicates that the ALD‐Al_2_O_3_ layer can suppress the Sn loss during the sulfurization. **Supporting** **Table** **S3:** Compared elemental ratios of the sulfurized AZTS‐a‐CZTS films with different pre‐sulfurization temperature for the cosputtered Cu‐ZnS‐SnS precursor. The data is measured by ICP‐OES. **Supporting** **Table** **S4:** Photovoltaic parameters and Suns‐*V*_OC_ results of the representative solar cells from the AZTS‐a‐CZTS samples with different pre‐sulfurization temperature at 400°C (400‐AgAl), 420°C (420‐AgAl) and 440°C (440‐AgAl). The *n*_eff_, *J*_01_ and *J*_02_ are extracted from the Suns‐*V*oc results, representing effective junction diode factor, recombination current in bulk and recombination current at space charge region, respectively. **Supporting** **Table** **S5:** Photovoltaic parameters of the representative solar cells with different coating cycles of AZTS on pre‐sulfurized CZTS. **Supporting** **Table** **S6:** Photovoltaic parameters of the representative AZTS‐CZTS and AZTS‐a‐CZTS samples with postdevice annealing (PA).

## Funding

This work was supported by Australian Centre of Advanced Photovoltaics (RG172864‐B, RG200768‐A), Australian Research Council (DE230100021, DP230102463, FT190100756, LP200301593).

## Conflicts of Interest

The authors declare no conflicts of interest.

## Supporting information

Supplementary Material

## Data Availability

The data that support the findings of this study are available from the corresponding author upon reasonable request.
