# Supplementary material for: Device Postannealing with Superficially Ag‐Modified Absorber for High‐Efficiency Cd‐Free Cu2ZnSnS4 Solar Cells
Source: Small Sci. 2026 Feb 21;6(2):e202500545. doi: 10.1002/smsc.202500545 (PMC12928119; doi:10.1002/smsc.202500545)
Supplement: Supplementary file 1 — Supplementary Material [file SMSC-6-e202500545-s001.pdf]

Supporting information

Device post-annealing with superficially Ag-modified absorber for high-efficiency Cd-free Cu_2ZnSnS_4 solar cells

Xiaojie Yuan, Jialiang Huang, Jianjun Li, Kaiwen Sun, Ao Wang and Xiaojing Hao[*]

X. Yuan, Dr. J. Huang, Prof. J. Li, Dr. K. Sun, A. Wang and Prof. X. Hao*

School of Photovoltaic and Renewable Energy Engineering, University of New South Wales, Sydney, NSW 2052, Australia

Prof. J. Li

Shenyang National Laboratory for Materials Science, Institute of Metal Research, Chinese Academy of Sciences, Shenyang, Liaoning, 110016, China

E-mail: xj.hao@unsw.edu.au

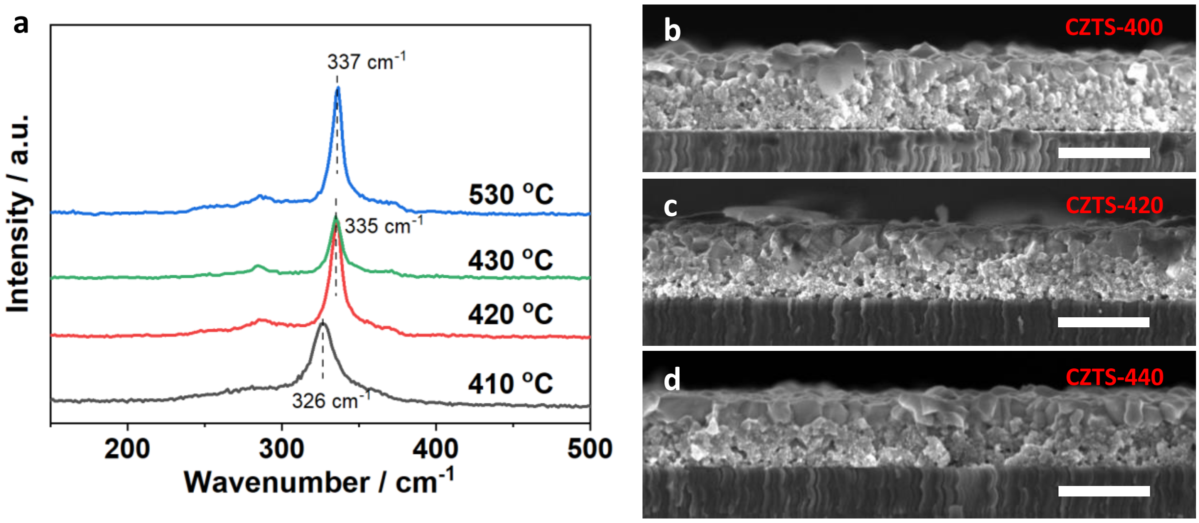

Figure S1. (a) Raman spectra of pre-sulfurized CZTS films at different temperature for 2 min. The film forms wurtzite CZTS phase at 410 °C (326 cm^{-1}) and transforms into kesterite phase at over 420 °C (335-337 cm^{-1}), cross-sectional view SEM images of pre-sulfurized co-sputtered Cu-ZnS-SnS precursor at different pre-sulfurization temperatures: (b) 400 °C, (c) 420 °C and (d) 440 °C. The unit of the scale bar is 1 µm.

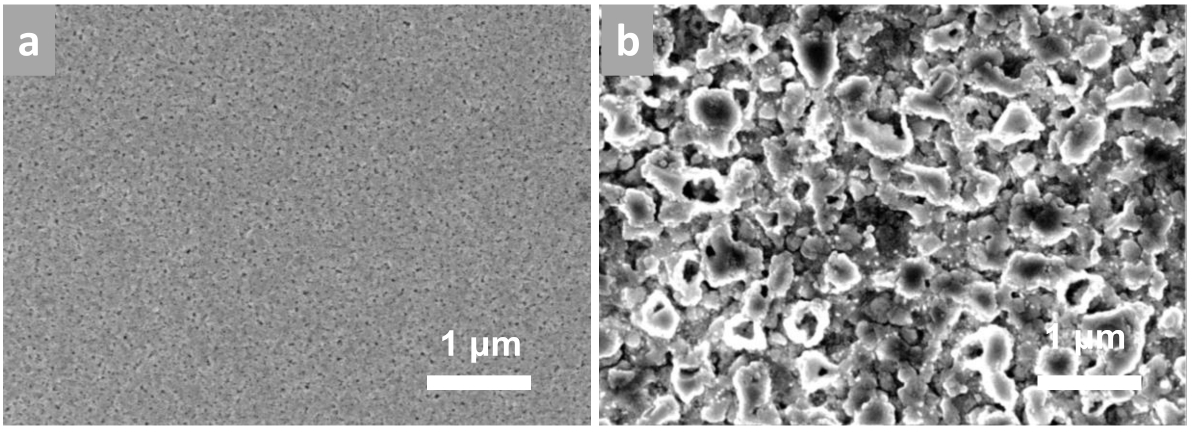

Figure S2. top-view SEM images of AZTS layer coated on (a) Mo and (b) pre-sulfurized CZTS film at 400 ºC.

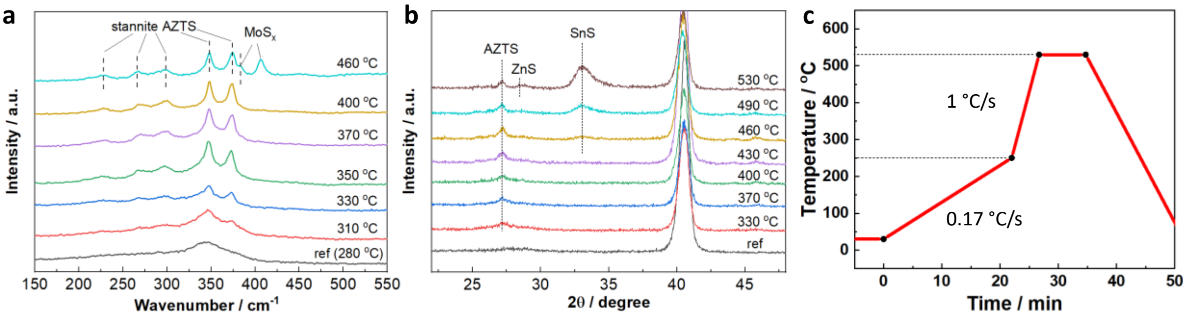

Figure S3. (a) Raman spectra and (b) XRD pattern of AZTS films on Mo after annealing in N_2 atmosphere at different temperature. Here we use three layers of AZTS spin-coated films instead of the single layer employed on pre-sulfurized CZTS film to increase the signal collection from Raman and XRD measurement. Raman spectra show the peaks of stannite AZTS phases (marked in dashed lines) start to appear at around 310 °C during the annealing, while the XRD pattern shows the spin-coated AZTS suffers from measurable phase segregation (AZTS, ZnS and SnS) at between 430 ºC and 460 ºC. (c) typical annealing profile for the sulfurization of CZTS precursor films.

Table S1 Compared elemental ratios of AZTS films annealed at different temperatures in N_2 atmosphere. The data is measured by SEM-EDS. The comparable Ag/Sn ratios at 370 ºC and 400 ºC and increased Ag/Sn ratio from 2.045 at 400 ºC to 2.161 at 430 ºC indicates that the Sn loss (in form of SnS as depicted in Figure S3b) and decomposition of the AZTS film start at between 400 ºC and 430 ºC.

SEM-EDS	Ag/Sn	Zn/Sn
AZTS-370	2.052	1.426
AZTS-400	2.045	1.482
AZTS-430	2.161	1.491
AZTS-460	2.191	1.373

Table S2 Compared elemental ratios of reference and AZTS-modified CZTS films after sulfurization. The data is measured by ICP-OES. The lower (Ag+Cu)/Sn ratio of AZTS-a-CZTS absorber at 1.896 than that of AZTS-CZTS absorber at 1.918 indicates that the ALD-Al_2O_3 layer can suppress the Sn loss during the sulfurization.

Sample	Ag/(Cu+Ag)	(Ag+Cu)/Sn
CZTS	0.000	1.896
AZTS-CZTS	0.031	1.918
AZTS-a-CZTS	0.033	1.896

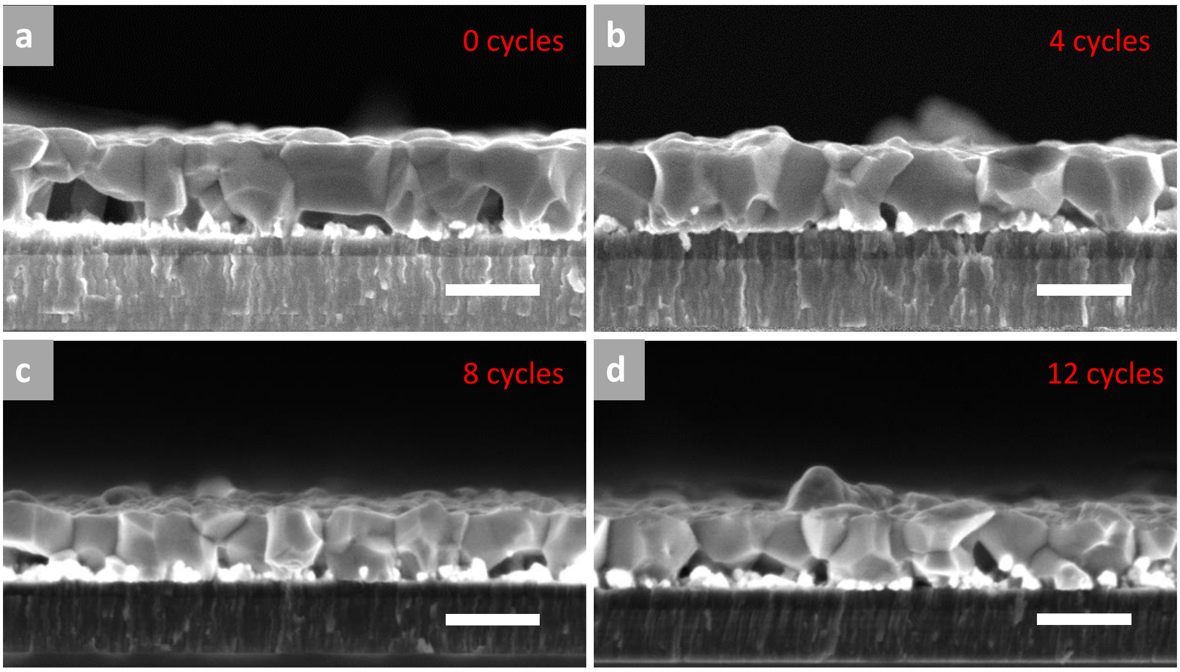

Figure S4. Cross-sectional SEM images of sulfurized AZTS-a-CZTS films with different cycles of ALD-Al_2O_3: (a) 0, (b) 4, (c) 8, and (d) 12. The unit of the scale bar is 1 μm. The ALD-Al_2O_3 starts to limit the grain growth with 8 cycles by introducing more horizontal grain boundaries near the surface.

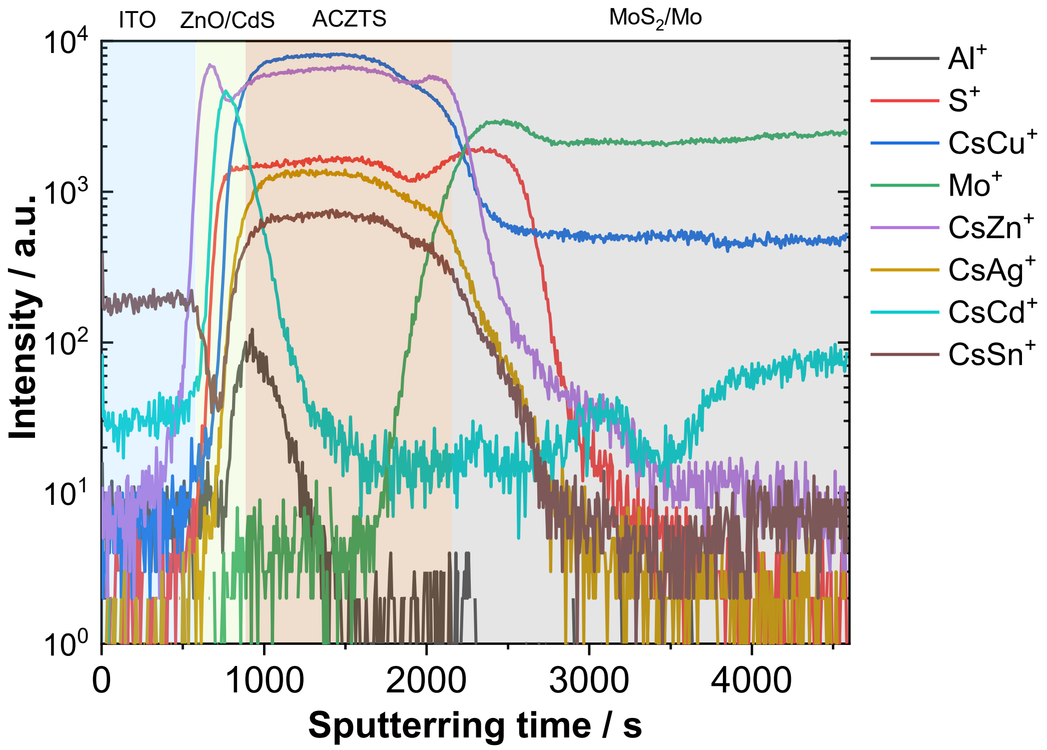

Figure S5. SIMS depth profile of the typical AZTS-a-CZTS device with CdS buffer layer.

Table S3 Compared elemental ratios of the sulfurized AZTS-a-CZTS films with different pre-sulfurization temperature for the co-sputtered Cu-ZnS-SnS precursor. The data is measured by ICP-OES.

samples	(Ag+Cu)/Sn
CZTS	1.896
AZTS-a-CZTS-400	1.908
AZTS-a-CZTS-420	1.896
AZTS-a-CZTS-440	1.853

Table S4 Photovoltaic parameters and Suns-V_{OC} results of the representative solar cells from the AZTS-a-CZTS samples with different pre-sulfurization temperature at 400 ºC (400-AgAl), 420 ºC (420-AgAl) and 440 ºC (440-AgAl).

	J_{SC} (mA cm^{-2})	V_{OC} (mV)	FF (%)	Eff. (%)	R_{sh} (Ω cm^2)	R_s (Ω cm^2)	E_g (eV)	E_g/q-V_{OC} (V)	n_{eff}	J_{01} (A cm^{-2})	J_{02} (A/cm^{-2})
400-AgAl	18.69	704	60.53	7.97	468	1.32	1.56	0.856	1.97	2.48×10^{-13}	6.36×10^{-7}
420-AgAl	19.25	722	60.04	8.35	524	0.69	1.57	0.848	2.00	1.58×10^{-13}	5.90×10^{-7}
440-AgAl	19.16	706	56.82	7.69	458	1.28	1.59	0.884	2.02	1.18×10^{-13}	9.10×10^{-7}

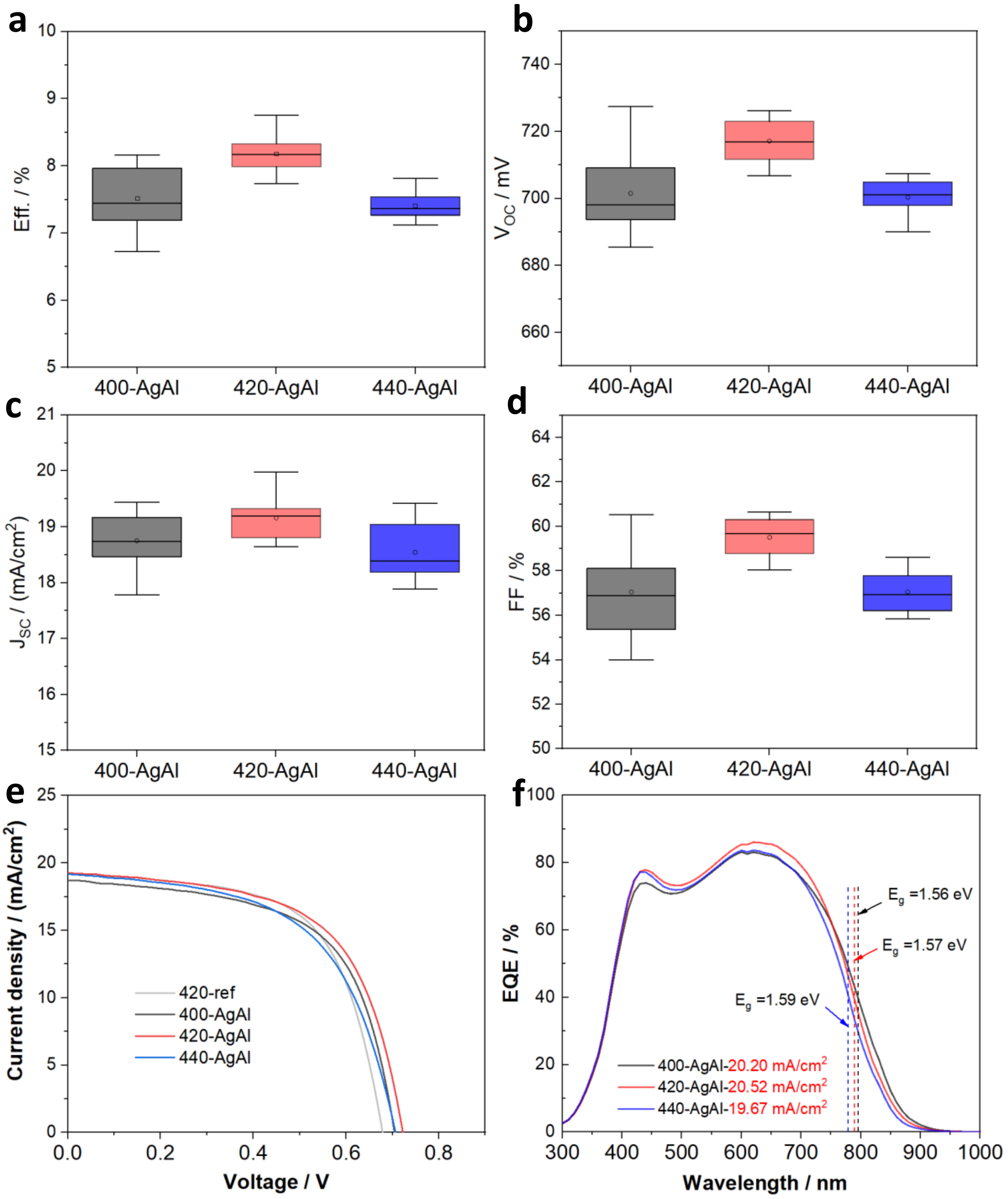

Figure S6. Comparison of the statistical distribution of the device performances of AZTS-a-CZTS samples with different pre-sulfurization temperatures for the co-sputtered Cu-ZnS-SnS precursor at 400 °C (400-AgAl), 420 °C (420-AgAl) and 440 °C (440-AgAl): (a) efficiency, (b) V_{OC}, (c) J_{SC}, (d) FF. The sample size is 12 cells for both samples which are measured based on total area device. (e) representative J-V curves and (f) EQE response of the AZTS-a-CZTS samples with different pre-sulfurization temperatures. The corresponding band gaps derived from d(EQE)/dλ and integral J_{SC} are given in Figure S6f.

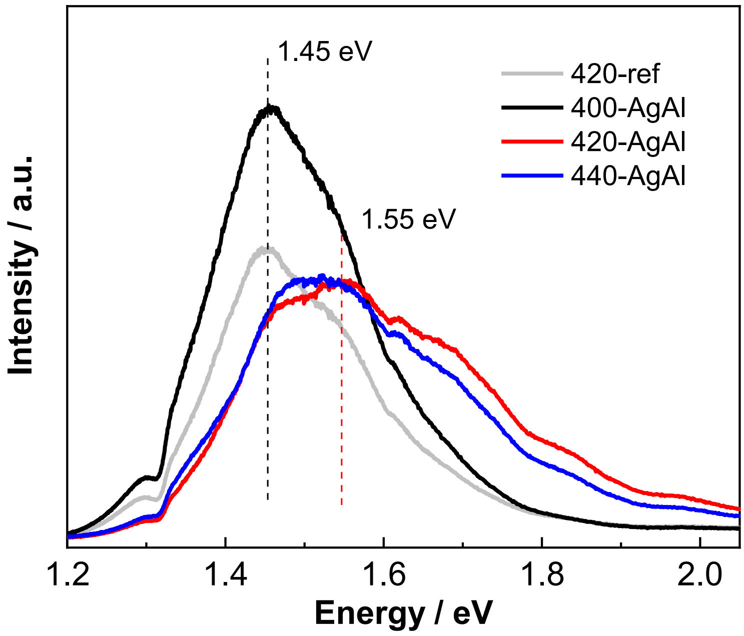

Figure S7. PL spectra (λ_{ex} = 525 nm) of the reference CZTS and AZTS-a-CZTS devices with different pre-sulfurization temperatures at 400 ºC (400-AgAl), 420 ºC (420-AgAl) and 440 ºC (440-AgAl).

Table S5 Photovoltaic parameters of the representative solar cells with different coating cycles of AZTS on pre-sulfurized CZTS.

	J_{SC} (mA cm^{-2})	V_{OC} (mV)	*FF* (%)	Eff. (%)
One-layer AZTS	18.95	711	61.15	8.24
Two-layer AZTS	18.25	699	57.54	7.34

Note S1: Calculation method of Urbach energy (E_U)

The E_U can be roughly calculated via the EQE data in Figure 5f, where the E_U can be obtained from the following equation:

$$\ln[-ln(1 - EQE)] = lnA + \frac{h\nu}{E_U}$$

The E_U can be obtained from a plot of ln[-ln[1-EQE]] vs. $h\nu$ converted from the EQE spectra, where the slopes $1/E_U$ are linearly fitted within the photon energy slightly below the bandgap derived from the EQE spectra.

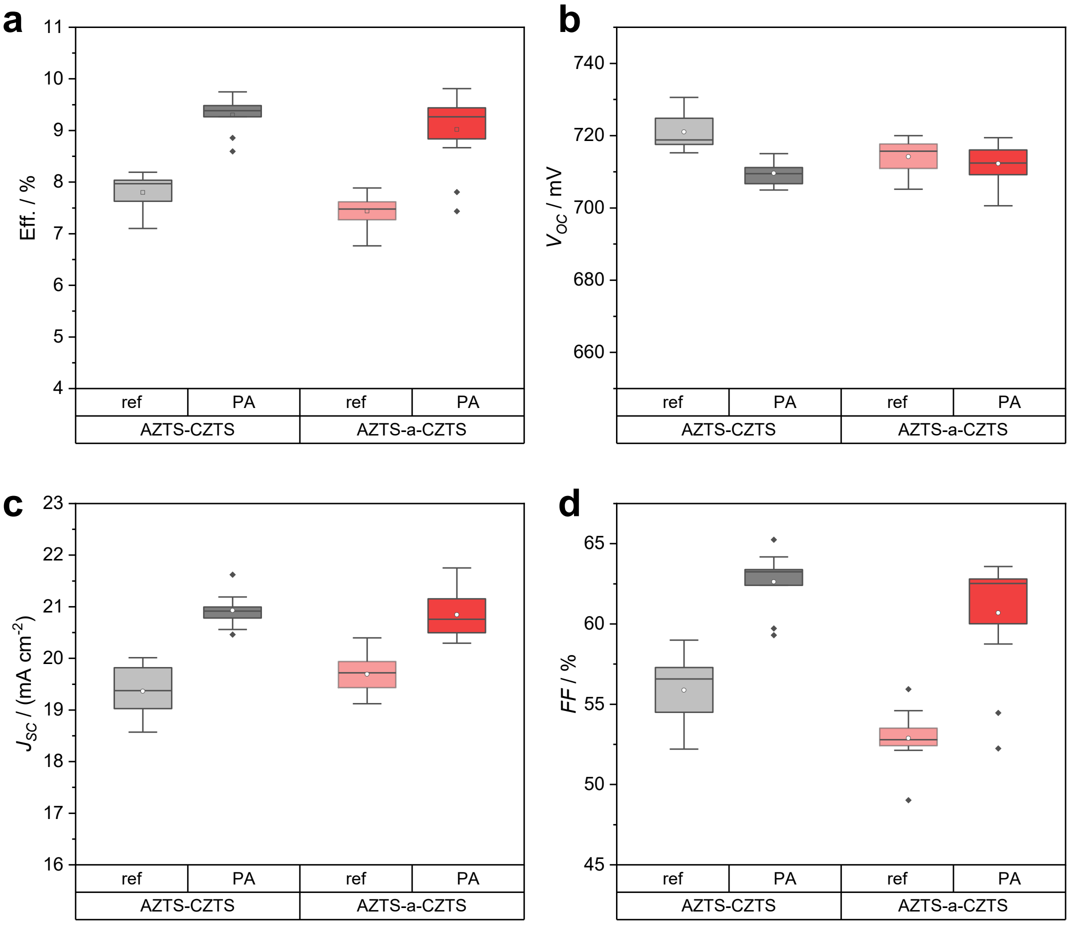

Figure S8. Comparison of the statistical distribution of the device performances of AZTS-CZTS and AZTS-a-CZTS samples with and without PA: (a) efficiency, (b) V_{OC}, (c) J_{SC}, (d) FF. The sample size is 12 cells for both samples which are measured based on total area device.

Table S6 Photovoltaic parameters of the representative AZTS-CZTS and AZTS-a-CZTS samples with post-device annealing (PA).

	J_{SC} (mA cm^{-2})	V_{OC} (mV)	FF (%)	Eff. (%)	R_{sh} (Ω cm^2)	R_s (Ω cm^2)	A	J_0 (A cm^{-2})	E_g (eV)	E_g/q-V_{OC} (V)
AZTS-CZTS-PA	21.19	705	65.25	9.75	759	0.44	2.26	3.14×10^{-6}	1.53	0.825
AZTS-a-CZTS-PA	20.93	714	62.91	9.41	821	0.81	2.88	3.08×10^{-6}	1.54	0.826

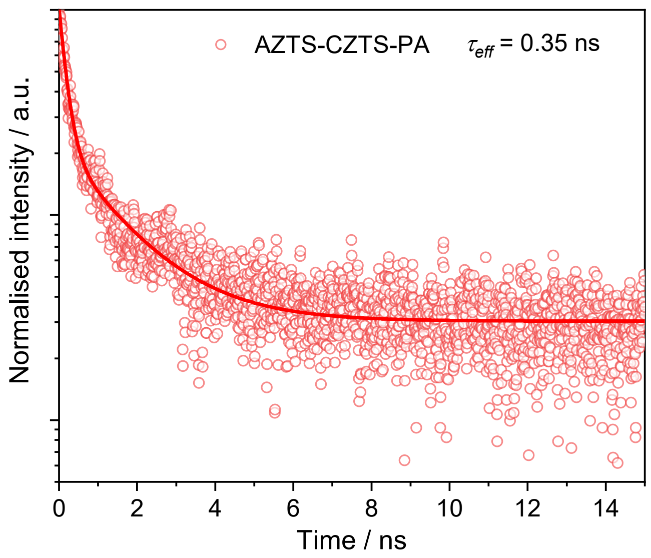

Figure S9. TRPL (λ_{ex} = 640 nm) decay of the AZTS-CZTS devices after PA process with calculated effective lifetime (τ_{eff}) from double exponential function.
